# Transcatheter tricuspid valve interventions: Current status and future perspectives

**DOI:** 10.3389/fcvm.2022.994502

**Published:** 2022-09-14

**Authors:** Alberto Alperi, Marcel Almendárez, Rut Álvarez, Cesar Moris, Victor Leon, Iria Silva, Daniel Hernández-Vaquero, Isaac Pascual, Pablo Avanzas

**Affiliations:** ^1^Heart Area, Hospital Universitario Central de Asturias, Oviedo, Spain; ^2^Cardiac Pathology Department, Health Research Institute of Asturias (Instituto de investigación Sanitaria del Principado de Asturias), Oviedo, Spain; ^3^Department of Medicine, Faculty of Medicine, University of Oviedo, Oviedo, Spain

**Keywords:** transcatheter tricuspid valve repair, transcatheter tricuspid valve replacement, tricuspid regurgitation, valve heart disease, tricuspid incompetence

## Abstract

The development of transcatheter therapies to treat valvular heart diseases has changed the rules of the game, particularly in the case of aortic stenosis and mitral regurgitation. In the last years, the tricuspid valve has also been the focus of these percutaneous techniques for several reasons: (1) tricuspid regurgitation is a frequent disease associated with poor clinical outcomes in spite of medical treatment, leading to end-stage right ventricular heart failure, (2) surgical tricuspid valve repair or replacement has been the treatment of choice for patients with tricuspid valve disease, but high mortality rates for isolated surgical tricuspid valve intervention have been reported in the literature, and (3) most patients with tricuspid pathology are ultimately denied cardiac surgery because of their comorbidity burden. Thus, in this context the development of less invasive catheter-based therapies would be of high clinical relevance. The present review provides an overview regarding the framework of chronic tricuspid regurgitation transcatheter therapeutic options, summarizes the transcatheter systems under clinical use or clinical evaluation with their technical features, and describes the updated current evidence in this challenging and evolving field.

## Introduction

Tricuspid regurgitation (TR) has been demonstrated to be the 2nd most frequent regurgitant valvular heart disease in the United States, only surpassed in prevalence by mitral regurgitation (1). Additionally, owing to the steadily increased in life expectancy observed for patients exhibiting left-sided valve disease and in those ones with ventricular dysfunction there will likely be an increment in the prevalence of TR over the upcoming years.

The etiology of TR is mainly functional for the vast majority of patients seen in clinical practice (2), usually associated with left heart pathologies leading to pulmonary hypertension and/or right chambers' dilation. Although primary (organic) TR is quite infrequent in western countries, a growing number of cases exist of pacemaker or implantable defibrillator-induced TR owing to the interference between the leads and the valve leaflets (3).

Significant TR has been associated with poor clinical outcomes in several cardiac settings (4, 5) and, if left untreated, it may lead to gradual annular and right ventricular (RV) dilatation and ultimately end-stage RV heart failure. Historically, surgical tricuspid valve repair or replacement has been the treatment of choice for patients with tricuspid valve disease. Currently, the European guidelines for the management of patients with valvular heart disease recommend surgery with the greatest class of recommendation for patients with severe TR undergoing left-sided valve surgery. However, for patients with isolated severe TR, the class of recommendation drops to IIa (should be considered). This statement applies to all severe TR patients regardless of a prior left-sided valve intervention, although a meticulous consideration of distinctive features that increase the surgical risk, such as RV or left ventricular dysfunction and pulmonary hypertension, is mandatory (6). The 2020 ACC/AHA guidelines for the management of valve heart disease are in line with the European ones, although they differentiate between patients with severe secondary TR with or without a prior left-sided valve surgery: a IIb (could be considered) class of recommendation for tricuspid surgery is given for the former, whereas the latter receive a IIa (should be considered) class (7).

However, it should be noted that several patients with tricuspid pathology are ultimately denied cardiac surgery because of their comorbidity burden alongside with their frequently advanced heart failure status. Additionally, the mortality rates for isolated surgical tricuspid valve interventions have been relatively high (close to 10%) in several series (8, 9), and the paucity of robust data translates into the absence for tricuspid specific surgical risk score assessment according to the Society of Thoracic Surgeons (STS) score, in opposition to their mitral and aortic counterparts. A novel score for assessing surgical risk in isolated tricuspid valve disease has been developed recently, and its performance is to be evaluated over the following years. According to the surgical series from which the score stemmed, the majority of patients had at least a 5% risk for early post-surgical mortality (10).

Hence, over the last decade an increasing number of transcatheter therapies have been developed aiming to fulfill this unmet clinical need.

In the present review we aimed to outline the current challenges of the transcatheter tricuspid valve intervention (TTVi) field, highlight the main considerations for TTVi planning and guiding, and provide an updated view of the current TTVi dedicated systems under clinical evaluation along with their technical characteristics and main results. TTVi using replacement systems intended for the aortic valve for treating patients with prior tricuspid annuloplasty and tricuspid bioprosthetic dysfunction are beyond the focus of this review.

## Tricuspid valve anatomy: Considerations for TTVi

The tricuspid is the largest of the heart valves. It lays caudal and is one of the most easily accessible valves for transcatheter interventions as it does not require neither arterial access nor septal crossing (11). However, the tricuspid valve system has some specific challenges for transcatheter interventions: (i) it has complex subvalvular components including multiple chordae tendinae and papillary muscles, increasing the risk of entrapment and hindering device maneuverability; (ii) the annular size is D-shaped, dynamic over the cardiac cycle and highly dependent on intravascular volume and hemodynamic conditions; (iii) the close anatomic relationship between the septal tricuspid annulus and the atrioventricular node and His system pose a risk for new-onset or worsening conduction disturbances, especially in orthotopic replacement scenarios; (iv) the mid and distal segments of the right coronary artery reside in the atrioventricular groove, laying in proximity (<3 mm at some points) to the tricuspid annulus, what increases the risk for acute coronary compression in annuloplasty and replacement cases; and (v) the short space between the inferior vena cava (IVC) opening into the right atrium (RA) and the tricuspid valve makes the appropriate coaxial positioning of the intended systems more challenging, as they need to acutely bend within a relatively small cavity.

Overall, the tricuspid position has several unique characteristics to be considered for transcatheter procedures.

## Preprocedural workup and procedural guidance

The severity, etiology and mechanisms of TR should be carefully assessed. Besides, the cardiac imaging examination should help determine patient eligibility in accordance with the characteristics of every specific device, to inform on the risk of potential complications, and to evaluate the likelihood of successfully abolish or at least significantly reduced TR.

### Echocardiography

Pre-procedural transthoracic echocardiography (TTE) is the first cardiac imaging exam to be performed, as it enables to assess RV size and function and to rule out any significant left-sided valve disease. However, transoesophageal echocardiography (TEE) is the cornerstone technique for procedural guidance in both repair and replacement procedures, and requires close collaboration between the sonographer and the interventional team. A list of commonly used features for TEE planning and guidance in TTVi procedures is presented in Table 1. Biplane images taken from the mid or deep esophagheal 60–80° commissural view help determine the origin of the main jet(s) to be treated, and 3D surgical-like views enable a safe approach and bending of the guiding catheter and delivery systems on their way toward the targeted valve. During the procedure, multiple mid-esophagheal views along with short-axis transgastric projections are paramount for navigating and orientating purposes. Additionally, TEE allows for the diagnosis of immediate complications (e.g., paravalvular leakage, cardiac tamponade, device malposition, etc.) and for the early assessment of the success of the intervention in terms of TR reduction and residual diastolic gradients. Specific guidelines for the assessment and quantification of TR (12), as well as for a step-by-step guidance in several TTVi settings (13) are available elsewhere.

**Table 1:** Basic features concerning TEE and TTVi.

**Pre-procedural TEE**	**Intra-procedural TEE**
Degree of TR and presence of tricuspid stenosis. Quality of the TEE imaging for procedural guidance with the patient in the supine position. Coexisting of non-tricuspid valve disease. Annular dimensions and leaflet length.	**Annuloplasty repair**: -3D TEE atrial surgical-like view and multiplane 2D views to assess the position of the anchors. - Assess annular depth from the hinge point of the leaflet. -Evaluate distance from the RCA.
Morphologic characterization and classification of tricuspid leaflets. Location of targeted jet origin and evaluation of coaptation defect(s). Rule out right chamber thrombus and lead-related complications.	**Edge-to-edge repair**: -Bi-plane sweeping views from the 60° mid esophagheal to assess trajectory, position relative to the target jet and leaflet grasping. - Transgastric short-axis view for assessment of rotation and perpendicularity.
Subvalvular apparatus characterization: calcification, number, and position of chordae tendineae.	**Replacement**: -High-support guidewire localization in the tricuspid annulus. -System coaxiality and depth.
Rule out thrombus.	Final hemodynamic assessment: residual TR, tricuspid gradients.

### Cardiac computed tomography

**Figure 1:**
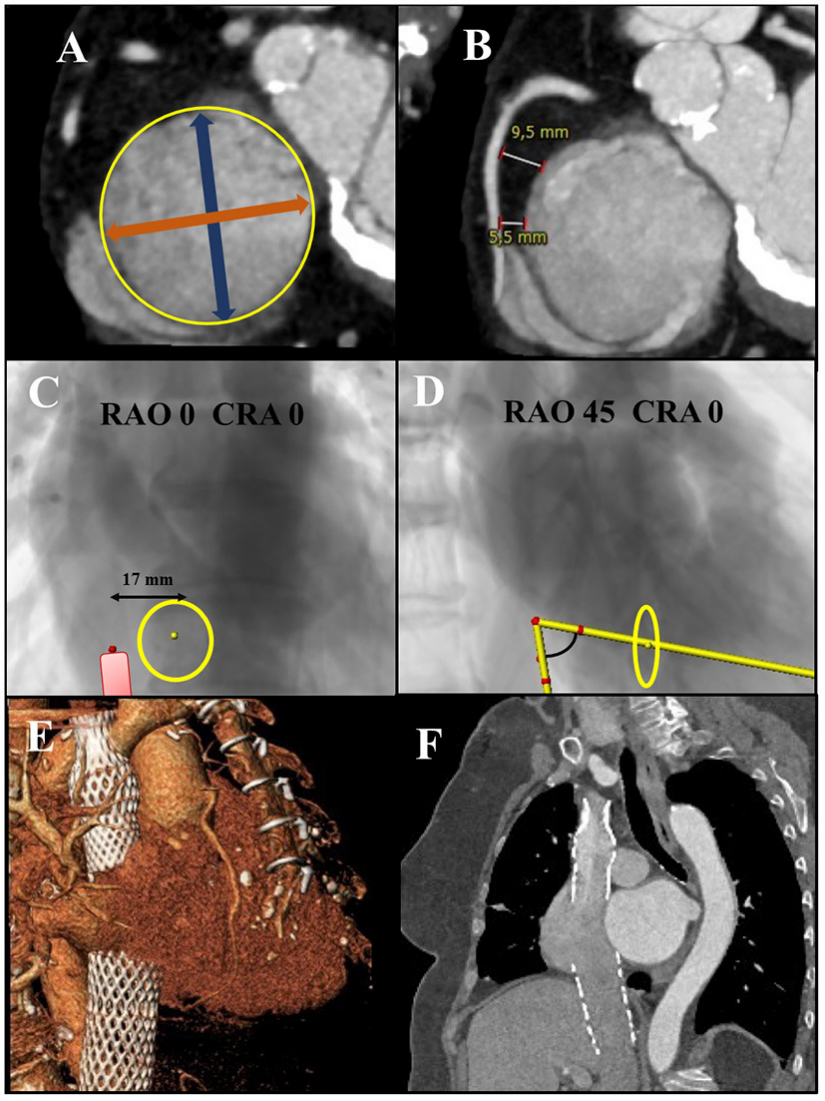
**(A)** CCT reconstruction for the assessment of tricuspid annular sizing before TTV annuloplasty repair or TTV replacement. Yellow circle represents tricuspid annular perimeter, blue double-headed arrow the anteroposterior diameter, and orange double-headed arrow the septo-lateral diameter. **(B)** CCT reconstruction assessing the distance between the tricuspid annulus and the right coronary artery. **(C)** Antero-posterior fluoroscopic view of the CCT-derived IVC-RA junction to mid-tricuspid annular distance. **(D)** Fluoroscopic right anterior oblique view demonstrating the angulation to be undertaken by the device delivery system. **(E)** CCT 3-dimensional reconstruction after Tric-valve implantation. **(F)** CCT coronal view after Tric-valve implantation.

Despite the important amount of information obtained from TEE, cardiac computed tomography (CCT) is considered to be essential for most of the TTVi procedures, with the exception of edge-to-edge repair. CCT enables for millimetric image resolution, hence facilitating an accurate assessment of several landmarks: the tricuspid annular dimensions over the cardiac cycle (paramount for annuloplasty systems and orthotopic replacement) (Figure 1), the space between the tricuspid annulus and the right coronary artery in short-axis images, the distance between the tricuspid annular plane and the right ventricular apex (useful in some coaptation systems), the number and position of chordae tendinae and papillary muscles (planning of bouquet-like repair systems), the sizing of the vena cavae close to their junction within the RA (cornerstone for sizing in heterotopic replacements), and the distance between the vena cavae and RA junction to the mid tricuspid annulus in coronal planes (the larger the distance, the greater the bend of the guiding catheter for proper alignment) (Figure 1). Several specific sizing algorithms are used for the variety of replacement systems available nowadays, each of them taking into consideration different anatomical points.

## Devices with clinical data: Transcatheter tricuspid valve repair

The currently used TTV repair systems are summarized in Tables 2, 3 according to their mechanism of action. Specific considerations for every device are summarized as follows.

**Table 2:** Baseline and procedural characteristics and 30-day outcomes of patients undergoing TTV repair with edge-to-edge devices.

	**Edge-to-edge repair**					
	**MitraClip**			**TriClip**	**PASCAL**	
	**Besler et al**.	**Mehr et al**.	**Ruf et al**.	**Nickenig et al**.	**Fam et al**.	**Kodali et al**.
	**(14)**	**(15)**	**(16)**	**(17)**	**(18)**	**(19)**
*N* of patients	117	249	50	85	28	34
Age	79	79	80 (78–83)	77.8	78	76.3
STS-PROM score, %	5.3	NA	NA	NA	4.6 ± 2.8	7.3
Functional TR		223 (89)	NA	71 (84)		29 (88)
**TR severity**						
- None-/Moderate	7 (6)	8 (3)	7 (14)	5 (6)		1 (3)
- ≥Severe	110 (94)	241 (97)	43 (86)	80 (94)	28 (100)	32 (97)
**Procedural outcomes**						
Procedural success	95 (81)	192 (77)	50 (100)	76 (91)	24 (86)	24 (80)
Number of devices	2 ± 1	2 ± 1	1.7 ± 0.7	2.2 ± 0.8	1.4 ± 0.6	1.2
Conversion to open surgery	NA	1 (0.4)	0	0	0	NA
Device embolization or malposition	NA	NA	0	0	0	NA
**30-day outcomes**						
Mortality	0	7 (2.8)	0	0	2 (7.1)	0
Stroke	NA	2 (0.8)	NA	0	0	0
Major or life-threatening bleeding	NA	15 (6)	NA	0	0	2 (5.9)
**TR severity**						
- None-Moderate	92 (78)	192 (77)	37 (54)	73 (85.6)	22 (85)	14 (52)
- ≥Severe	25 (22)	57 (23)	23 (46)	12 (14.4)	4 (15)	13 (48)

Continuous values are expressed as mean ± SD, mean (range) and median [interquartile range] as reported by authors. Categorical values are expressed as n (%).

**Table 3:** Baseline and procedural characteristics and 30-day outcomes of patients undergoing TTV repair with annuloplasty-like devices.

	**Annuloplasty-like devices**				**Chordae**
	**Cardioband**			**Trialign**	**Mistral**
	**Nickenig et al. (20)**	**Davidson et al. (21)**	**Nickenig et al. (22)**	**Hahn et al. (23)**	**Planer et al. (24)**
*N* of patients	30	30	61	15	7
Age	75.2	77	78.6	73.6	76
STS-PROM score, %	2.6	NA	7.1 ± 5.4	NA	5.3
Functional TR	30 (100)	30 (100)	61 (100)	15 (100)	7 (100)
**TR severity**					
- None-/Moderate	6 (24)	0	3 (6)	NA	0
- ≥Severe	19 (76)	30 (100)	50 (94)		7 (100)
Procedural success	NA	28 (93.3)	26/31 (83.9)	12 (80)	7 (100)
Number of devices	1	1	1	1	1.1
Conversion to open surgery	0	0	NA	0	0
Device embolization or malposition	0		1 (1.6)	1 (6.7)	0
Mortality	2 (6.7)	0	1 (1.6)	0	0
Stroke	1 (3.3)	0	0	0	0
Major or life-threatening bleeding	4 (13.3)	7 (23.3)	7 (11.5)	NA	0
TR severity				NA	NA
- None-Moderate	16 (76)	12 (45)	32 (59)		
- ≥Severe	5 (24)	15 (55)	22 (41)		

Continuous values are expressed as mean ± SD, mean (range) and median [interquartile range] as reported by authors. Categorical values are expressed as n (%).

TR, tricuspid regurgitation.

### Devices for leaflet repair

#### MitraClip

##### Device system and procedural points

**Figure 2:**
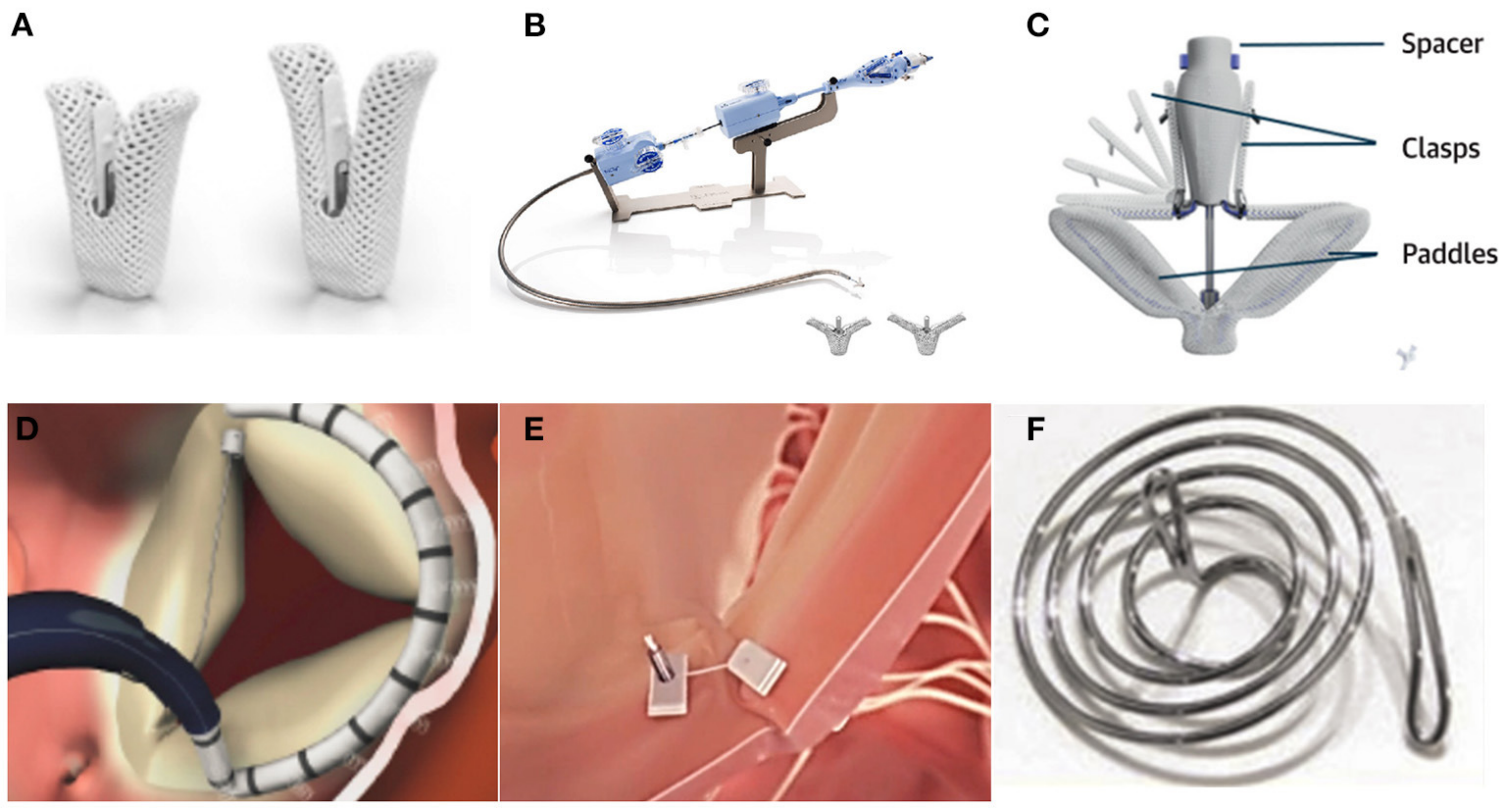
TTV repair devices under clinical evaluation. **(A)** TriClip device with standard and long-length arm [reprinted from Wong et al. (25) with permission from the publisher]. **(B)** TriClip delivery system. **(C)** PASCAL device [reprinted from (14) with permission from the publisher]. **(D)** Cardioband device [reprinted from (26) with permission from the publisher]. **(E)** Trialign device [reprinted from (22) with permission from the publisher]. **(F)** Mistral device [reprinted from (27) with permission from the publisher].

The MitraClip (Abbott, Menlo Park, Ca) device is a cobalt–chromium implant with two arms that are opened and closed with the use of the delivery-system handle (Figure 2A). The procedure is preformed *via* transfemoral vein, and the device is steered in the RA until correct alignment within the main jet origin is achieved. Then, the tricuspid leaflets are grasped and the device closed to approximate them. In its current version (G4 generation) 4 different types of MitraClip are available, either with 12-mm-length arms (XTr with a 4-mm width and XTw with 6-mm width) or 9-mm arms (NTr: 4-mm width; NTw: 6-mm width).

##### Current results

A few studies exclusively reported on the MitraClip utilization for the tricuspid position (14–16, 28, 29), and the main outcomes for the principal studies are displayed in Table 2. The rates of procedural success were systematically >75%, with a low rate of early complications. Early mortality and stroke rates were 2.8 and 0.8%, respectively.

##### Future perspectives

The purposely designed version of the MitraClip delivery system for the tricuspid position (TriClip) is expected to absorb the potential indications for Clip in the tricuspid valve.

#### TriClip

##### Device system and procedural points

The device itself is similar to the MitraClip. However, differential features exist at the level of the guiding catheter (implementation of a S/L knob allowing for septal and lateral bending and shortening of the curve to fit the RA-tricuspid valve angle) and delivery catheter (just a flexion/extension knob in contrast to the medial/lateral and anterior/posterior knobs of the mitral system) (Figure 2B). Both the guiding and delivery catheter are manipulated through the use of the aforementioned knobs until the Clip lays perpendicular to the line of coaptation at the intended point for grasping. Then, the device is advanced into the right ventricle and subsequently retrieved to grasp the leaflets.

##### Current results

Results from the single-arm TRILUMINATE trial both at 6-months (17) and 12-months of follow-up (26) have demonstrated a significant and sustained reduction in TR, with just 30% of the 63 patients with available 1-year data exhibiting a ≥ severe TR compared to 92% at baseline. Additionally, a significant decrease in the incidence of unplanned heart failure admission was found 1 year after procedure compared to the year before device implantation.

##### Futures perspectives

The randomized cohort of the TRILUMINATE trial (NCT03904147) will provide for the first time a clinical comparator to a transcatheter tricuspid intervention system. Patients will be randomized into TriClip implantation and optimal medical therapy vs. optimal medical therapy alone in a 1:1 basis. Besides, the CME approval of the TriClip device in Europe will open the door for real-life registries. The relatively high rates of single leaflet attachment (7% in the TRILUMINATE trial) are of particular concern, and future studies are eagerly awaited.

#### Pascal

##### Device system and procedural points

The PASCAL system consists of a 10 mm central spacer that acts as a filler in the regurgitant orifice along with 2 paddles and clasps. The paddles are broad (25 mm width) and the device can be elongated for safer maneuvering (Figure 2C). As the other edge-to-edge repair systems, it needs to cross the valve and grasp the tricuspid leaflets while pulling back, and all the steps and device positioning should be thoroughly monitored by intraprocedural TEE. The CLASP ACE version for this device is also available in clinical practice. In contrast to the PASCAL device, the PASCAL ACE has a smaller spacer as well as narrower paddles (6 vs. 10 mm) with more curvature. Overall, this results in a thinner profile for this device system which enables to capture more leaflet tissue relative to device size.

##### Current results

To date, data on the Pascal system for the tricuspid position stem from one observational study of compassionate-use cases in Europe (18) and the CLASP TR early feasibility study in North America (19). Overall, the procedure was safe and at least a 1-grade reduction was achieved for the vast majority of patients. However, close to half of the population remained with postprocedural TR ≥ severe in the feasibility trial.

##### Futures perspectives

The 2^nd^ study of the CLASP TR will randomize 825 subjects with at least severe and symptomatic TR to receive transcatheter repair with the Pascal system or medical therapy alone (NCT04097145). As for the Triluminate trial, it will provide for the 1^st^ time randomized data on the effects of TTVi with this leaflet approximation device.

### Devices for annular repair

#### Cardioband

##### Device system and procedural points

The implant consists of a contraction wire and polyester fabric covering with radiopaque markers attached to an adjustment mechanism (Figure 2D). It is designed to reduce TR *via* annular reduction, and it's affixed along the annulus of the valve using the 17 radiopaque anchors as a fluoroscopic reference.

After implantation at the anterior and posterior parts of the TA using fluoroscopy and transoesophageal echocardiography guidance, the device is cinched to reduce anteroposterior and septolateral tricuspid diameters.

##### Current results

Data from both the TRI-REPAIR (20) and the U.S. early feasibility study (21), with a total of 30 patients included in each of them, along with the TriBAND study (22) (*n* = 61) are currently available (Table 3). Besides, real-world data for this device has been recently published (27). The mortality rates ranged between 0 and 6.7%, although the rates of major bleeding events were relatively high (systematically > 10%). The degree of TR reduction varied across studies, although the vast majority of patients experienced at least a 1-grade reduction after device implantation. Recently, the 6-month outcomes of the early feasibility study were reported in a dedicated congress, with a 92% of overall survival, 19% reduction in annular dimensions and 78% of patients experiencing at least a 2-grade reduction in TR (30).

##### Futures perspectives

Besides the European TrIcuspid Regurgitation RePAIr With CaRdioband Transcatheter System (TRI-REPAIR) trial (NCT02981953) and the Transcatheter Repair of Tricuspid Regurgitation With Edwards Cardioband TR System Post Market Study (TriBAND) post-market study (NCT03779490), a US-based study evaluating early feasibility is under way (NCT03382457).

#### Trialign

##### Device system and procedural points

TrialignTM device (Mitralign, Inc.) represents a novel percutaneous tricuspid valve annuloplasty technique that requires a double access *via* the right jugular vein. During the Trialign procedure a pair of polyester pledgets are delivered across the tricuspid annulus in proximity to the anteroposterior and septo-posterior commissure, cinched by a polyester suture to obliterate the posterior tricuspid leaflet, and locked on the atrial side (Figure 2E). First, a radiofrequency guidewire is used to cross the tricuspid annulus from the ventricular into the atrial chamber at the septo-posterior portion of the tricuspid annulus. Then, the guidewire is snared through the 2^nd^ access and the 1^st^ pledget install. The 2^nd^ device requires the same technique and it's positioned at the antero-posterior commissure. Finally, a dedicated plication lock delivery catheter is advanced over both pledget sutures to the atrial side of the tricuspid annulus, facilitating the plication of the portion of the tricuspid annulus in between the pledgets.

##### Current results

Results of the Early Feasibility of the Mitralign Percutaneous Tricuspid Valve Annuloplasty System Also Known as Trialign (SCOUT) trial (23) demonstrated that all 15 patients underwent successful device implantation with no serious complications but for an ST elevation myocardial infarction due to plication of the right coronary artery requiring stent implantation. A total of 3 patients (20%) had single pledget detachment at 30-day echocardiographic follow-up. At least a 1-grade TR reduction was observed for the as-treated population (patients without detachment).

##### Futures perspectives

An early feasibility trial is ongoing in China (NCT04936802), and the SCOUT-II trial (NCT03225612) will evaluate the safety and performance of this device on 60 patients with functional TR.

### Devices for chordal repair

#### Mistral

##### Device system and procedural points

The device consists of a delivery system and a spiral-shaped single nitinol wire, and it's been built to enable an atraumatic grasping of the chordae tendineae, forming a “flower bouquet” shape that approximates the chordae and the leaflet (Figure 2F). The procedure is performed *via* the femoral vein using an 8.5F steerable catheter and a delivery system. Once at the RA the device is advanced through the center of the valve and navigated to the target commissure, and then rotated clockwise to capture the chordae.

##### Current results

The first in human feasibility study has been recently reported, demonstrating a great safety profile of the device without major procedural or 30-day complications. Additionally, all patients experienced at least a 1-grade reduction in TR after procedure, translating into a significant reduction in vena contracta and effective regurgitant orifice area, and that was accompanied by an improvement in all RV function parameters (24).

##### Future perspectives

The MATTERS (NCT04071652) and MATTERS II (NCT04073979) trials will further evaluate the clinical outcomes of this promising device in patients with severe symptomatic functional TR.

#### Other devices

The TriCinch system (a stainless-steel corkscrew placed in the anterior annulus with a self-expanding nitinol stent deployed at the IVC) was tested in a series of 24 patients, and the results were presented at a dedicated congress (31). The rate of procedural success was 75%, and 2 (8%) and 4 (17%) cases of hemopericardium and annulus detachment, respectively, were of concern. This system is no longer under clinical use.

The FORMA repair system is a foam-filled polymer balloon that expands once positioned across the tricuspid valve. A single-arm trial demonstrated the feasibility of this procedure, although a significant proportion of cases (>60%) remained with significant TR (32, 33). This device system is no longer under clinical use.

The DaVingi device (Cardiac Implants, Tarrytown, New York) consists of a ring delivery system and an annuloplasty ring. A first-in-human report was recently published (34), and a first-in-human study, which is planning to include 15 patients, is currently ongoing (NCT03700918).

The Millipede IRIS device is semi-rigid nitinol ring with 8 independent anchors that works as a complete annuloplasty ring. Its development focuses on the mitral position, although a dedicated tricuspid delivery system is in progress.

The transatrial intrapericardial tricuspid annuloplasty (TRAIPTA) is a procedure in which a circumferential device is deployed within the pericardial space to decrease tricuspid annular dimensions. Pericardial access is gained through the right atrial appendage, and the system aims at encircle the atrioventricular groove (35).

The pledget-assisted suture tricuspid annuloplasty (PASTA) technique creates a double-orifice tricuspid valve *via* a double pledgeted polyester suture which is percutaneously attached to the tricuspid annulus. The first-in-human procedure was reported, although the patient presented annular deshicence needing further intervention (36).

## Devices with clinical data: Transcatheter tricuspid valve replacement

Current TTV replacement systems are summarized in Table 4.

**Table 4:** Baseline and procedural characteristics and 30-day outcomes of patients undergoing TTV replacement.

	**Orthotopic valves**				**Heterotopic valves**	
	**Navigate**	**Evoque**		**LuX-Valve**	**Sapien XT**	**Tricento**
	**Hahn et al. (37)**	**Fam et al. (38)**	**Webb et al. (39)**	**Lu et al. (40)**	**Dreger et al. (41)**	**Schaefer et al. (42)**
*N* of patients	30	25	132	12	14	27
Age	75 ± 10	76 ± 3	79 ± 7	69 (66–74)	75 ± 8	73 ± 10
STS-PROM score, %	NA	9.1 ± 2.3	5.3 ± 4.3	NA	NA	NA
Euroscore II	11.1% (7.1–14.1%)	7.7 ± 2.2	7.4 ± 5.39	NA	NA	NA
Functional TR	NA	19 (76)	93 (70.5)	NA	NA	NA
**TR severity**						
- None-/Moderate	2 (6.6)	4 (16)	13 (12)	0	0 (0)	NA
- ≥Severe	28 (92.4)	21 (84)	119 (88)	12 (100)	14 (100)	
**Procedural outcomes**						
Procedural success	26 (87)	23 (92)	128 (96)	12 (0)	14 (100)	30 (96.1)
Conversion to open surgery	2 (6.6)	0	NA	NA	4 (28)	NA
Device embolization or malposition	0	0	NA	0 (0)	4 (28)	1 (3)
**30-day outcomes**						
Mortality	3 (10)	0	4 (3.2)	1 (8)	3 (21)	NA
Stroke	1 (3.3)	0	0 (0)	0	NA	NA
Major or life-threatening bleeding	10 (30)	3(12)	2 (2)	1 (8)	3 (21)	NA
**TR severity**						
- None-Moderate	18 (76)	23 (92)	NA	11 (92)	NA	NA
- ≥Severe	6 (24)	2 (8)	NA	1 (8)	NA	NA

Continuous values are expressed as mean ± SD, mean (range) and median (interquartile range) as reported by authors. Categorical values are expressed as n (%).

TR, tricuspid regurgitation.

### Orthotopic bioprosthesis

#### The gate system

##### Device system and procedural points

**Figure 3:**
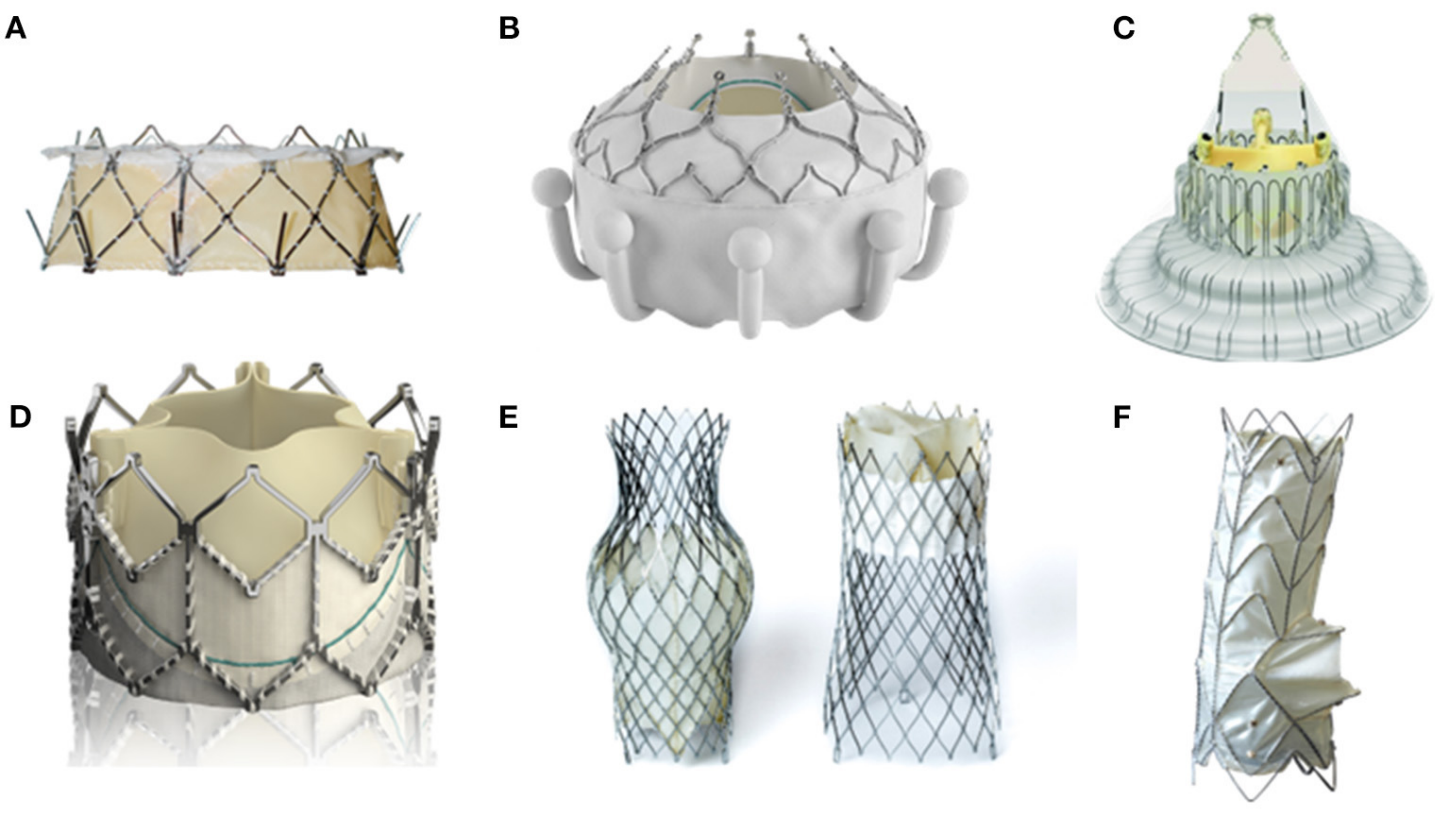
TTV replacement devices under clinical evaluation. **(A)** Gate System; **(B)** Evoque Valve; **(C)** LuX-Valve; **(D)** Sapien XT valve; **(E)** TricValve; **(F)** Tricento system. Reprinted with permission from the publisher.

The GATE (NaviGate Cardiac structures Inc., Lake Forest, California) system is a self-expanding bioprosthesis composed of a nitinol alloy conical stent with three pericardial leaflets that is implanted *via* jugular access using a 42-F introducer (Figure 3A) (37, 43, 44).

##### Current results

A single cohort of 30 patients reported an 87% success rate, with only 2 cases requiring conversion to open-heart surgery. Patients presented a reduction ≥ 2 grades of regurgitation with 76% with mild or less TR at discharge. In-hospital mortality was 10%. After a follow-up of 127 ± 82 days, 62% of the cases were in NYHA functional class I or II and four additional deaths (13%) were reported (37).

##### Future perspectives

Further studies with a larger population to allow proper patient selection to reduce complications are warranted with no clinical trials underway.

#### Evoque

##### Device system and procedural points

The Evoque valve (Edwards Lifesciences, Irvine, California) is a self-expanding nitinol frame valve with bovine pericardial leaflets which is delivered through a 28 F transfemoral approach (Figure 3B) (38, 39).

##### Current results

A 25-patient observational study showed a technical success of 92%. There were no conversions to surgery and no procedural deaths. After 30-days, 76% were at NYHA I-II functional class, and over 90% presented a grade I-II TR with a 0% mortality rate (38). The results from the TRISCEND II study are encouraging, with sustained mild or less TR at 12-months in 96% of the cases with 93% free from HF hospitalization and a 93% survival rate (45).

##### Future perspectives

The ongoing TRISCEND II (NCT04482062) multicenter, randomized control trial compared to OMT will conclude its first phase by 2024.

#### LuX-Valve

##### Device system and procedural points

The LuX-Valve (Ningbo Jenscare Biotechnology Co., Ningbo, China) is a trileaflet bovine pericardium valve. It is delivered through a minimally invasive thoracotomy and subsequent transatrial approach using a 32F steerable system (Figure 3C) (40, 46).

##### Current results

A compassionate multicenter study of 46 patients with severe TR not suitable for surgery showed a success rate of 97.8%. After 6-months of follow-up, mortality was 17,4%, with one case of device migration. Mild or less TR was reported in 80% of the patients (46).

##### Future perspectives

The TRAVEL study (NCT04436653) is a prospective multicenter single-arm open trial to recruit 150 patients to evaluate the safety and effectiveness of the LuX-Valve.

#### Other devices

The Cardiovalve (Cardiovalve Ltd., Or Yehuda, Israel) is a **s**elf-expanding nitinol frame valve including three bovine pericardial leaflets (47). Current clinical evidence is scarce since the valve is in its early stages, with a few clinical cases showing favorable outcomes at 2 years (47).

The Intrepid valve (Medtronic, MN, USA), intended for the mitral space, has been successfully used on the tricuspid valve in some case reports (48). It is a dual stent bovine pericardial valve that can be successfully retrieved at any time before the final release. The valve is introduced *via* a 35F transfemoral access. A feasibility trial is ongoing.

The Trisol valve (Trisol Medical, Haifa, Israel) was designed explicitly for the tricuspid valve with available published results only for animal models. A first-in-human study has been approved.

### Heterotopic bioprosthesis

Caval valve implantation (CAVI) reduces the systolic backflow into the caval veins, subsequently reducing right heart failure symptoms and volume overload. It is a choice for specific anatomies where orthotopic valves are not suitable (e.g., severe annular dilation and previous pacemaker) (49).

#### Sapien

##### Device system and procedural points

The Sapien valve is a balloon-expandable device designed to for the aortic position. It has been used off-label for TR treatment.

##### Current results

The TRICAVAL trial randomized a total of 28 patients to OMT or CAVI, with the study being stopped due to an unexpectedly high rate of valve dislocations (28.5%) with no significant differences regarding symptoms and exercise capacity (Figure 3D) (41).

##### Future perspectives

The ongoing HOVER trial is enrolling high-risk inoperable patients and will hopefully shed some light on adequate patient selection for this procedure.

#### Tricvalve

##### Device system and procedural points

The Tricvalve is a specifically designed bicaval self-expanding system. A 30 mm tubular stent for the superior vena cava is inserted through the jugular vein. The inferior vena cava valve is inserted through the femoral vein, requiring a 27F access (Figure 3E) (49, 50).

##### Current results

Clinical experience is limited to case reports for compassionate use. Aparisi et al. reported a significant improvement in the NYHA class and 6-min walking test (51). Similarly, Lauten et al., in the first in-human case, showed immediate hemodynamic improvement with abolition of the ventricular wave in the IVC and improved NYHA functional class (52).

##### Future perspectives

There are currently two randomized controlled trials. The TRICUS (NCT03723239) feasibility study based in the United States will enroll 10 patients and its European counterpart, the TRICUS Euro (NCT04141137), 35 patients. The main objective includes serious adverse events in the first 30-days and NYHA class at 6-months.

#### Tricento

##### Device system and procedural points

The Tricento valve is a single bicaval stent with a lateral bileaflet bovine pericardial valve. It is inserted through a 24F delivery system through the femoral vein and implanted from the SVC toward the IVC (Figure 3F) (53).

##### Current results

Compassionate use for 31 cases were reported in the TCT 2020, registering a 96.7% success rate (42). Cruz-Gonzalez et al. evaluated the system in 6 patients and after a mean follow-up of 11 months, all the patients showed improvement in ≤ II NYHA class (54). Conversely, Wilbring et al. noticed recurring signs of right heart failure, with magnetic resonance showing a nearly full systolic stent compression at the right atrium (55).

##### Future perspectives

There are no current ongoing trials for this system owing to problems with the patency of the stent.

#### Other devices

The CroiValve DUO consists in a coaptation device made of soft pericardial tissue that is delivered across the tricuspid annulus *via* the right jugular vein. It enhances leaflet coaptation and its central valve facilitates diastolic filling. Finally, the system is anchored to the supervisor vena cava by means of a stent. A few human implants have already been performed (56).

## Clinical perspectives

### Overview of the clinical results

Overall, the early experience with TTVi has yielded low rates of early procedural-related complications along with relatively high rates of procedural success (systematically above 70%). Although major bleeding rates have been close to those reported in the early experience of dedicated repair/replacement systems for the mitral position (57), the rates of stroke events have been systematically lower in the tricuspid field. This fact may be mainly explained by the lack of transseptal puncture and left atrial navigation and manipulation, hence allowing for safer procedures in terms of cerebrovascular complications. This fact is of paramount important in a frail, elderly and comorbid population in which a great percentage of patients do need chronic oral anticoagulation and exhibit prior cerebrovascular disease. There is certainly a trade-off between stroke events and pulmonary embolic complications, although the latter are way less meaningful in terms of mortality and quality of life.

The amount of TR reduction has been variable among studies and devices, and remain (especially for transcatheter tricuspid repair) as one of the major caveats to be improved over the upcoming years. Although the vast majority of patients had at least a 1-grade reduction in TR after the intervention, it should be noted that many of them remained with at least moderate TR despite a successful procedure. The clinical impact of these otherwise incomplete TR repairs is yet to be established. The edge-to-edge leaflet repair field accounts for the largest experience among TTVi techniques, and it has been demonstrated that a large leaflet coaptation gap is an independent predictor for device success in this setting (16). In fact, coaptation gaps > 10 mm have been systematically excluded from single-arm trials with latest-generation devices (17, 19). This anatomical issue should be carefully considered for proper patient selection.

Most of the studies have demonstrated a benefit in patients' clinical status after TTVi. Nevertheless, the studies reported thus far lacked a comparator (i.e., a control group not undergoing tricuspid intervention), and whether the systematic improvement in NYHA functional class leads to any survival benefit needs to be proven. Besides, it must be outlined that most of the studies reported exclusively on early outcomes, and mid- and long-term findings for these devices are largely unknown. Therefore, the current results should be taken cautiously, and future trials comparing TTVi using several devices vs. optimal medical therapy, as well as long-term follow-up results for the ongoing studies will shed more light on this matter.

### Future prospects: Transcatheter tricuspid valve repair or replacement

**Figure 4:**
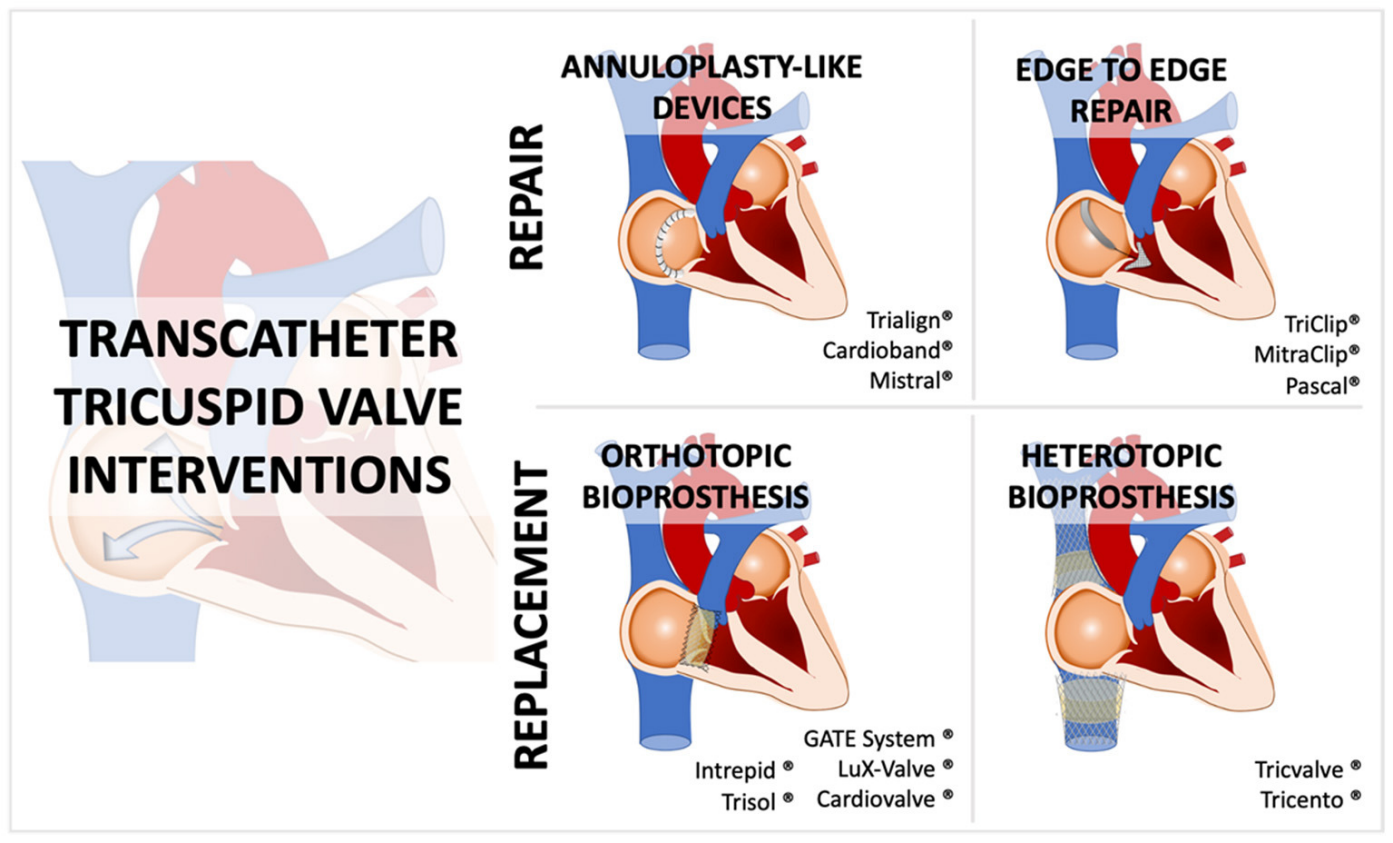
Overview of the several devices and their mechanism of action in the transcatheter tricuspid valve intervention field.

The tool-box for the percutaneous treatment of tricuspid valve disease is rapidly expanding (Figure 4), and a trend toward a tailored device selection taking into consideration anatomical features, clinical status and patients' risk profile is expected in the near future. A recent meta-analysis focusing on observational and single-arm trials for transcatheter tricuspid valve repair systems has demonstrated extremely low rates for major cardiovascular complications, especially for the edge-to-edge technique, whereas the main caveat for percutaneous tricuspid repair interventions seems to be the relatively high rates of residual significant TR (58). Therefore, current data suggest the superiority of TTV replacement vs. repair regarding valve performance and the presence of residual TR. On the other hand, TTV replacement lies way behind repair in terms of procedural safety and major adverse cardiovascular events. Albeit TTV replacement might be more effective regarding complete TR resolution (“surgical-like” result), its use should be balanced against the aforementioned pitfalls in a case-by-case scenario. Anatomical aspects preventing optimal repair should always be assessed, and in such cases (e.g., coaptation gaps > 7 mm, pacemaker leads precluding optimal repairment, or severely restricted leaflets combined with calcification) a replacement approach may stand out as the upfront strategy. However, given its safety profile, a repair procedure may be favored in doable cases in which the interventional team anticipates great possibilities for at least a 2-grade reduction in TR and/or a residual TR regurgitation grade ≤ moderate.

Another controversial aspect is the potential need for chronic oral anticoagulation in patients with a prosthetic valve in the tricuspid position. Although less data exists on tricuspid prosthetic valve replacement compared to their mitral counterparts, it is broadly assumed that the blood stasis observed in the lower-pressure right atrial chamber may easily lead to prosthetic valve thrombosis. As a consequence, chronic oral anticoagulation seems mandatory for TTV replacement recipients. On the contrary, the smaller frame size utilized in repair systems do not require upfront anticoagulation. Although most of the patients receiving TTVi present with atrial fibrillation, this frail and largely comorbid patient population often need bridging anticoagulation strategies due to non-cardiac interventions, ultimately increasing the risk for bleeding and thrombotic (including prosthesis-related) events. Direct oral anticoagulants seem a good alternative in these clinical scenarios, although their performance in the setting of transcatheter heart valves is debatable (59, 60) and no study to date has evaluated their clinical use for prosthesis in the tricuspid position. Long-term data on device durability and valve thrombosis with current replacement therapies will be of upmost importance.

In regards to heterotopic TTV replacement, it must be considered that these interventions aimed exclusively to reduce congestion and its consequences. However, given that TR is not a direct target for this therapy, progressive RV dilation and dysfunction will likely not be tackled, and this poses a great shortcoming for this alternative.

Studies providing direct head-to-head comparisons between transcatheter tricuspid repair vs. replacement systems are not expected in the near future. Therefore, a close monitoring of clinical and echocardiographic outcomes for studies reporting on either of both techniques are pivotal for enhancing our understanding of these therapies. Meanwhile, an individual approach after a comprehensive evaluation of anatomical and clinical characteristics by the Heart Team seems mandatory. Optimal patient selection is key for continuous improvement in the transcatheter tricuspid field.

## Conclusion

A variety of transcatheter devices exist nowadays aiming to treat tricuspid regurgitation either by means of repair or replacement. The early results are promising for these systems, associating low rates of procedural-related complications and high procedural success. However, several issues such as residual tricuspid regurgitation with repair systems, valve durability and valve thrombosis with replacement therapies, and overall clinical impact on hard mid- and long-term outcomes need further investigation.

## Author contributions

Concept and design and writing the article: AA, MA, IP, and PA. All authors contributed to the critical revision of the article and approved the submitted version.

## Conflict of interest

The authors declare that the research was conducted in the absence of any commercial or financial relationships that could be construed as a potential conflict of interest.

## Publisher's note

All claims expressed in this article are solely those of the authors and do not necessarily represent those of their affiliated organizations, or those of the publisher, the editors and the reviewers. Any product that may be evaluated in this article, or claim that may be made by its manufacturer, is not guaranteed or endorsed by the publisher.
